# A New Strategy for Rapid Diagnosis of the Source of Low Back Pain in Patients Scheduled to Undergo Treatment with Cooled Radiofrequency Ablation

**DOI:** 10.3390/diagnostics11101822

**Published:** 2021-10-01

**Authors:** Shih-Hsiang Chou, Cheng-Chang Lu, Sung-Yen Lin, Po-Chih Shen, Zi-Miao Liu, Wei-Hsing Chih, Chia-Lung Shih

**Affiliations:** 1 Department of Orthopedics, Kaohsiung Medical University Hospital, Kaohsiung Medical University, Kaohsiung 807, Taiwan; stanelychou@gmail.com (S.-H.C.); cclu0880330@gmail.com (C.-C.L.); tony8501031@gmail.com (S.-Y.L.); shenporch@gmail.com (P.-C.S.); 2 Orthopaedic Research Center, Kaohsiung Medical University, Kaohsiung 807, Taiwan; 3 Graduate Institute of Medicine, College of Medicine, Kaohsiung Medical University, Kaohsiung 807, Taiwan; 4 Regenerative Medicine and Cell Therapy Research Center, Kaohsiung Medical University, Kaohsiung 807, Taiwan; 5 Department of Orthopedics, Kaohsiung Municipal Siaogang Hospital, Kaohsiung 812, Taiwan; 6 College of Medicine, Kaohsiung Medical University, Kaohsiung 807, Taiwan; p21actin@yahoo.com.tw; 7 Department of Orthopedics, Ditmanson Medical Foundation Chia-Yi Christian Hospital, Chia-Yi City 600, Taiwan; 8 Clinical Medicine Research Center, Ditmanson Medical Foundation Chia-Yi Christian Hospital, Chia-Yi City 600, Taiwan

**Keywords:** low back pain, ^99m^Tc-MDP SPECT/CT, a modified finger test, radiofrequency ablation

## Abstract

Objective: The objective of this study was to develop a new strategy for rapid diagnosis of the source of low back pain (LBP) for treatment with cooled radiofrequency ablation (RFA). Materials: Patients suffering from facet joint (FJ) or sacroiliac joint (SIJ) pain for more than 3 months were included. Two methods, Technetium Tc99m methylene diphosphonate single photon emission tomography/computed tomography (^99m^Tc-MDP SPECT/CT) and a modified Fortin finger test were used to identify the source of LBP for treatment with cooled RFA. The visual analog scale (VAS) and Oswestry disability index (ODI) were used to assess the patients’ pain levels and disabilities respectively. These two measures were recorded at baseline and 1-week, 1-month, 3-month, and 6-month follow-up visits. Results: A total of 40 patients with LBP were included in this study. Our results demonstrated that the patients with LBP identified by our new strategy had significant improvements in VAS or ODI score at 1-week to 6-month follow-up visits (*p* < 0.001) after receiving cooled RFA. Similar results were also found in patients with FJ pain and those with FJ and SIJ pain respectively. Among all the patients, over 70% had greater than or equal to 50% reduction in VAS and ODI scores. No serious adverse events were observed after treatment. Conclusions: This new strategy could be successfully adopted for rapid diagnosis of the source of comprehensive LBP.

## 1. Introduction

Low back pain (LBP) is one of the most common diseases affecting about 12% of Taiwanese adults [1] and is the leading cause of disability worldwide [2]. LBP incurs a substantial economic burden which is estimated to exceed $100 billion dollars annually for the treatment of this disease in the United States [3]. The potential sources of LBP of the spinal column are soft tissue, disc degeneration, and joints related to nociplastic pain. In addition, the major sources of LBP are lumbar facet joints (FJ) (21–41% of patients) [4] and sacroiliac joints (SIJ) (16–30% of patients) [5]. Achieving a definitive diagnosis for patients with LBP is difficult, and the treatment is not always effective [6]. LBP has been treated to date with traditional methods, such as intra-articular steroid injections [7]. Neurotomy is a more recent alternative treatment for LBP, such as radiofrequency ablation (RFA) [8]. A recent meta-analysis demonstrated that the efficacy of RFA for treating LBP was significantly better than conservative treatment [8].

RFA is a minimally invasive surgery in which RF generates thermal energy to ablate the sensory nerve fibers of the FJ or SIJ for pain relief. Three RFA techniques have been developed for the treatment of LBP, including cooled, pulsed, and thermal RFAs [9,10]. These RFA techniques demonstrated significant improvements in LBP for up to 12 months [11]. Among these RFA techniques, cooled RFA is a relatively novel technique, and its probes produce larger lesions than the other two techniques and thus the efficacy of cooled RFA is expected to be better than the other two RFAs. A recent meta-analysis has demonstrated that the efficacy of cooled RFA seems to be better than pulsed and thermal RFAs [11].

For RFA treatment, diagnostic nerve blocks are the most commonly used method for identifying the specific source of LBP [12]. The underlying principle of this method is that the pain relief is tested by using local anesthesia to block the affected joint nerve [12,13]. A positive response indicates that the joint has a high possibility of being the source of pain [13]. The procedure requires performing the test on two different occasions, with at least 80% relief of familiar pain intensity on the two occasions being considered as a positive response [14]; however, performing the test on two different occasions for identifying the source of LBP is quite time-consuming, so it is critical to develop a more efficient method for rapid diagnosis of the source of LBP.

Technetium Tc99m methylene diphosphonate (^99m^Tc MDP) bone scan activity is an imaging marker of LBP. Bone scan activity on ^99m^Tc MDP single photon emission tomography/computed tomography (^99m^Tc-MDP SPECT/CT) can be used to identify the specific source of LBP [15]. It is a relatively rapid method for identifying the source of LBP; however, its diagnostic accuracy is reported as being only 72% compared with diagnostic nerve blocks [16]. Otherwise, another rapid method for identifying the source of LBP, the Fortin finger test, has been developed [17]. It is a simple and reliable method in which the source of LBP is localized by patients with one finger [17]. However, the Fortin finger test is specifically for SIJ pain [17], and in this study, we attempted to modify the Fortin finger test for identifying both FJ and SIJ pain. The combination of ^99m^Tc-MDP SPECT/CT and our modified finger test to identify the source of LBP might increase diagnostic accuracy for LBP.

The objective of this study was to develop a new strategy (combination of ^99m^Tc-MDP SPECT/CT and a modified Fortin finger test) for rapid diagnosis of the source of LBP in the patient undergoing treatment with cooled RFA. This novel diagnosis strategy is expected to benefit the patients with LBP by allowing them RFA faster than by identifying the source of the LBP using diagnostic nerve blocks.

## 2. Materials and Methods

### 2.1. Patients

This cohort study was approved by the Institutional Review Board (IRB) of the Kaohsiung Medical University Hospital (IRB number: KMUHIRB-E(II)-20200372). The patients with LBP receiving cooled RAF between March 2019 and June 2020 were retrospectively evaluated. The inclusion criteria were as follows: (1) patients suffering from low back pain for more than 3 months; (2) low back pain originating from facet or sacroiliac joints; (3) age ranging from 30 to 90 years; and (4) complete clinical outcomes for all follow-up visits. The exclusion criteria were as follows: (1) patients with known or suspected malignancy, infectious diseases, trauma, chronic inflammatory diseases, and (2) patients that were <30 or >90 years of age.

### 2.2. Source of LBP Diagnosis

Two methods were used to identify the source of LBP, including ^99m^Tc-MDP SPECT/CT and a modified Fortin finger test. The source of LBP identified by either of the two methods was considered a positive response.

#### 2.2.1. Modified Fortin Finger Test

The study referenced the Fortin finger test and provided a modified version [17], where one observer asked patients to point to the region of pain while standing and the observer localized the pain with a thumb. A source considered consistent over at least two trials was considered as a positive response and marked with a paper clip (Figure S1A). Moreover, the source of LBP was confirmed by a radiographic image.

#### 2.2.2. ^99m^Tc-MDP SPECT/CT

A previous study was referenced to perform ^99m^Tc-MDP SPECT/CT examinations [15]. Initially, patients received intravenous injections of 20 mCi (±10%) ^99m^Tc-MDP (800 MBq for those with age >75 years or weight >100 kg), then after 3 to 3.5 h of this protocol, patients were scheduled to undergo the ^99m^Tc-MDP SPECT/CT procedure (Figure S1B,C). All SPECT/CT examinations were performed on a Precedence 6-section or a 16-section scanner (Skylight SPECT system and Brilliance CT scanner; Philips Healthcare, Best, the Netherlands). The set of SPECT parameters was as follows: 128 × 128-word mode matrix, step-and-shoot angular step of 3°, 1.46 zoom factor, 128 views at 20 s per view, body contouring, and low-energy all-purpose collimator. The set of CT parameters was as follows: 60 mAs per section, 120 kVp, increment of 3 mm, and section thickness of 3 mm. Analysis specific to uptake activities in facet joints and sacroiliac joints were defined by a visual grading method (0 = nil activity, 1 = marginal activity, 2 = mild activity, 3 = mild to moderate activity, 4 = moderate activity, 5 = moderate to marked activity, 6 = intense activity) [18]. For scintigraphy, grades 0 to 2 were considered as negative and 3–6 as positive.

### 2.3. Cooled RFA Treatment

The treatment procedures of a previous study were followed to perform cooled RFA treatment [19]. All treatment procedures were conducted in a C-arm fluoroscopy suite (Figure S1D–F). During the treatment, patients received subcutaneously local anesthetic injection (0.5 mL of 2% lidocaine) and then an introducer was placed in the location. A cooled RFA SInergy probe (Kimberly–Clark Health Care, Roswell, GA, U.S.A.) was used for the dorsal ramus denervation. The local anesthetic was injected through the introducer for pain relief, after the correct electrode placement was identified. Then, a Pain Management Radiofrequency Generator (AVANOS* PMG115-Advance) was adopted to apply radiofrequency energy (set temperature = 60 °C; time = 2.5 min). Denervation of the medial branch for respective facet joints, or the medial branch of L5-S1 and concomitant S1, S2, S3 lateral branches for the respective sacroiliac joint were simultaneously performed. During the period of immediate post-operation, 0.5 mL of Betamethasone (RINDRON^®^; 4 mg/mL) was injected into each intervention point of spinal level targeted for pain relief.

### 2.4. Clinical Outcome Assessment

Up to date, there is no one method that can be used to identify the source of LBP accurately. Improvement in clinical outcomes seems to be an indicator to assess whether the source of LBP has been appropriately identified. Significant improvements in clinical outcomes indicate that the sources of LBP seem to have been identified accurately. Patients were followed up in our outpatient clinic at 1 week and 1, 3, and 6 months after receiving cooled RFA. Two measures were used to evaluate the clinical outcomes of patients at baseline and the four follow-up visits, including the visual analog scale (VAS) [20] and Oswestry disability index (ODI) [21]. The VAS was used to assess the patients’ pain levels during activity on a 10-cm line in which 0 cm indicated no pain and 10 cm indicated the worst pain. The ODI was used to assess the patients’ disability due to LBP and it includes ten items of disability (pain, personal care, lifting, sitting, standing, sleeping, sex life, social life, walking, and travelling). The score of each item ranges from 0 to 5, in which 0 indicates no functional limitation and 5 indicates a major functional disability. Thus, the maximum score of ODI is 50. The VAS was considered as primary outcome and the ODI was secondary.

### 2.5. Statistical Analysis

All statistical analyses were performed using IBM SPSS Statistics, version 19 (Armonk, NY, USA: IBM Corporation). Continuous variables were presented as mean and standard deviation. The changes between baseline and follow-up visits after adjusting for age, gender, and body mass index (BMI) were tested using the generalized estimating equations. A *p*-value less than 0.05 was considered to be of statistical significance.

## 3. Results

A total of 40 patients with LBP were included in this study. All of them had complete clinical outcomes at baseline and all the four follow-up visits, and completed the entire procedure smoothly without any sequelae (such as nerve root paresthesia and wound infections). The mean age of these patients was 63.05 ± 14.12 years and the mean BMI was 26.91 ± 4.60 kg/m^2^ (Table 1), with the majority being female (67.5%) (Table 1). A total of 16 patients were diagnosed with FJ pain and the other 24 patients were diagnosed with FJ and SIJ pain (Table 1).

**Figure 1:**
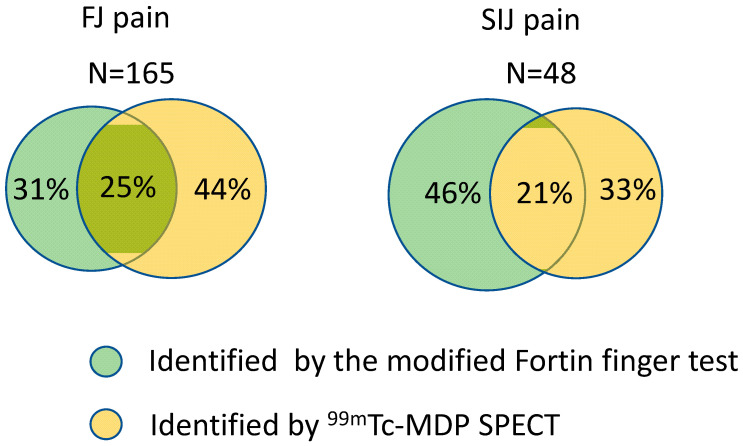
Overlap of sources identified by the modified Fortin finger test and ^99m^Tc-MDP SPECT for FJ and SIJ pain.

A total number of 165 sources of FJ pain were identified by ^99m^Tc-MDP SPECT or the modified Fortin finger test. Among these sources, 31% and 44% of them were only identified by ^99m^Tc-MDP SPECT and the modified Fortin finger test respectively, and only 25% of them were simultaneously identified by both methods (Figure 1). Otherwise, a total number of 48 sources of SIJ pain were identified by ^99m^Tc-MDP SPECT or the modified Fortin finger test. Among these sources, 46% and 33% of them were only identified by ^99m^Tc-MDP SPECT and the modified Fortin finger test respectively, and only 21% of them were simultaneously identified by both methods (Figure 1).

Although the mean of the initial VAS score of all the patients was 6.80 ± 0.91, these values were found to be 2.10 ± 0.93, 2.20 ± 0.76, 2.88 ± 0.76, and 3.28 ± 0.88 at the 1-week, 1-month, 3-month, and 6-month follow-up visits respectively. The improvements in VAS score became significantly better at 1-week (change = 4.70 ± 0.88, *p* < 0.001 vs. baseline), 1-month (change = −4.602 ± 0.93, *p* < 0.001 vs. baseline), 3-month (change = −3.93 ± 0.86, *p* < 0.001 vs. baseline), and 6-month (change = −3.53 ± 0.93, *p* < 0.001 vs. baseline) follow-up visits compared with the baseline level after adjusting for age, gender, and BMI (Table 2). The improvement in VAS score seemed to decrease with time (Figure S2a), with similar results also found in patients with FJ pain and those with FJ and SIJ pain respectively (Table 2 and Figure S2b,c).

Although the mean of the ODI scores of the patients was 36.03 ± 4.45 at baseline, these values were found to be 11.95 ± 3.85, 12.68 ± 3.55, 14.13 ± 2.86, and 16.45 ± 4.47 at the 1-week, 1-month, 3-month, and 6-month follow-ups respectively. After adjusting for age, gender, and BMI, the improvements in ODI scores became significantly better at 1-week (change = −24.08 ± 5.10, *p* < 0.001 vs. baseline), 1-month (change = −23.35 ± 5.60, *p* < 0.001 vs. baseline) 3-month (change = −21.90 ± 4.82, *p* < 0.001 vs. baseline), and 6-month (change = −19.58 ± 5.89, *p* < 0.001 vs. baseline) follow-up visits compared with the baseline level (Table 3). The improvements in ODI scores seemed to decrease with time (Figure S3a), with similar results also found in patients with FJ pain and those with FJ and SIJ pain respectively (Table 3 and Figure S3b,c).

**Figure 2:**
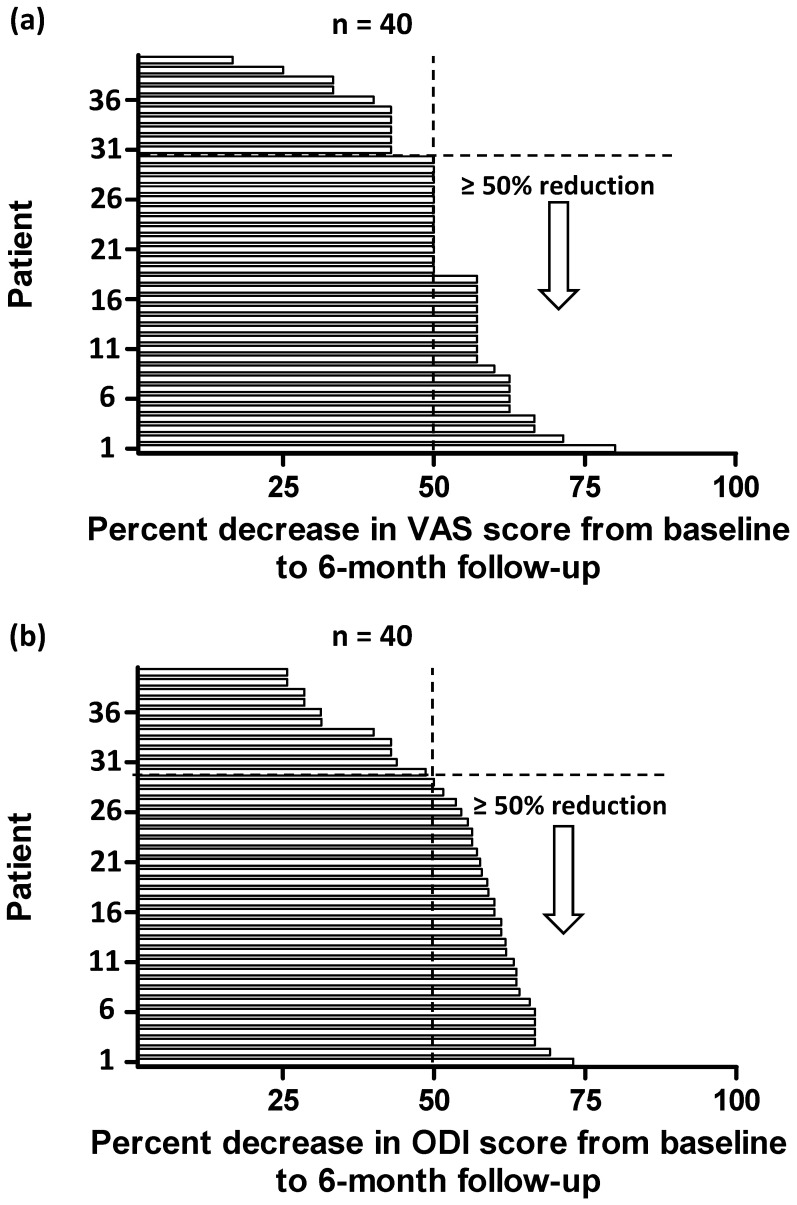
Individual responses in VAS (**a**) and ODI score (**b**) at 6 months after treatment, quantified by percent change from the baseline values.

Individual treatment responses from baseline to 6-month follow-up were calculated (Figure 2), and among these patients, 30 (75%) had ≥50% reduction in VAS score (Figure 2a), and 29 (73%) had ≥50% reduction in ODI score (Figure 2b); moreover, no serious adverse events were observed after treatment.

## 4. Discussion

Identifying the specific source of LBP is an important procedure for treatment with RFA, but current methods use much time or are specific for a certain type of LBP. Therefore, it is critical to develop a rapid-diagnostic method for identifying the source of comprehensive LBP. This study developed a new strategy (combination of ^99m^Tc-MDP SPECT and the modified Fortin finger test) for rapid diagnosis of the source of comprehensive LBP undergoing treatment with cooled RFA. Our results demonstrated that a relatively low percentage (21–25%) of sources was simultaneously identified by both methods for FJ or SIJ pain. These patients had significant improvements in VAS score from 1-week to 6-month follow-up visits compared with the baseline level; additionally, they also had significant improvements in ODI score from 1-week to 6-month follow-up visits compared with the baseline level. Similar results were also found in patients with FJ pain and those with FJ and SIJ pain respectively. In total, over 70% of patients had greater than or equal to 50% reduction in VAS and ODI scores, with no serious adverse events being observed after treatment. Our results seemed to indicate that our new strategy could be used to identify the source of LBP. Otherwise, the cost of our strategy is mainly from the examination using ^99m^Tc-MDP SPECT/CT which costs around US$239 [22]. However, two different occasions of diagnostic nerve blockers cost around US$1100 [23]. The cost of our strategy is much lower than that of diagnostic nerve blocks.

Pain relief after treatment with cooled RFA is the major concern of LBP. Our results demonstrated that the source of LBP identified by our new strategy had significant improvement in pain levels for up to the 6-month follow-up visit after receiving cooled RFA. Two previous studies using diagnostic nerve blocks to identify the source of LBP also reported similar results, with one of these demonstrating that patients with SIJ pain had significant improvement in pain levels over 6 months after treatment with cooled RFA [24], and the other indicating that patients with FJ pain had significant improvement in pain levels for up to 6 months after receiving cooled RFA [25].

In addition, a patient’s disability level after treatment with cooled RFA is also an important concern for LBP. Our results demonstrated that the patients receiving cooled RFA had significant improvement in ODI score for up to 6 months. A previous study using diagnostic nerve blocks to identify FJ pain also reported the same findings [25]. These results imply that using our new diagnostic strategy for identifying the source of LBP could cause significant improvements in pain levels and disability after receiving cooled RFA.

Based on a previous study that used cooled RFA to treat LBP, ≥50% reduction in NRS (numeric rating scale) pain score was defined as successful treatment [25]. They reported that a total of 52% of patients (11/21) had successful treatment at the 6-month follow-up visit [25]. Although we adopted the VAS score to assess patients’ pain levels, the VAS score is similar to the NRS score due to the same scale used for both types of score. Our results showed that 75% of patients (30/40) had ≥50% reduction in VAS score. Otherwise, ≥30% reduction in ODI score was defined as successful treatment in that study [25], where it was reported that a total of 62% of patients (13/21) had successful treatment at the 6-month follow-up visit [25]. Our results showed that 90% of patients (36/40) had ≥30% reduction in ODI score at 6 months. Our success rates were obviously higher than those of the previous study, which used diagnostic nerve blocks, indicating that our new strategy could be an alternative for identifying the source of comprehensive LBP.

The Fortin finger test has been adopted to identify the source only for SIJ pain [17]. However, we attempted to modify the Fortin finger test and our results showed that the modified Fortin finger test could identify not only SIJ pain but FJ pain as well, although the results demonstrated that a relatively low percentage (21–25%) of sources was simultaneously identified by both ^99m^Tc-MDP SPECT and the modified Fortin finger test for FJ or SIJ pain. This implies that the diagnostic accuracy of each method was relatively low. In combination, the two methods could increase high diagnostic accuracy and thus, a higher success rate in patients after receiving RFA could be achieved through this study.

There are some limitations in this study. Firstly, this was a retrospective cohort study without a control group. A high-quality randomized controlled trial should be further conducted to compare the efficacy between our new strategy and diagnostic nerve blocks for identifying source of LBP undergoing treatment with cooled RFA. Secondly, these patients were followed up for a short-term period, with the maintained efficacy being unclear for more than 6 months, and this would possibly decrease with time because of nerve regeneration after RFA [26]. It is not clear if the maintained efficacy of our strategy was the same as that of diagnostic nerve blocks. Thus, the efficacy of the use of our new strategy should be further confirmed for a longer-term period.

## 5. Conclusions

This study developed a new strategy for rapid diagnosis of the source of comprehensive LBP undergoing treatment with cooled RFA. The ^99m^Tc-MDP SPECT and a modified Fortin finger test were combined for identifying the source of LBP. This new strategy is easier to learn compared to diagnostic nerve block testing. Our results demonstrated that patients with LBP diagnosed by the new strategy showed significant improvements in pain and disability for up to 6 months after receiving cooled RFA, regardless whether the LBP is FJ pain or a combination of FJ and SIJ pain. The success rates of our study were obviously higher than those of the previous study, which used diagnostic nerve blocks only. These results indicate that our new strategy could be successfully adopted to identify the source of comprehensive LBP. This information can be used to develop a clinical prediction guideline for the use of these procedures. What was found here is cost effective and thus can change clinical practice if used where indicated.

## Figures and Tables

**Table 1:** Major characteristics of the patients at baseline.

Variable	All Patients	Patients with FJ Pain	Patients with FJ and SIJ Pain
n	40	16	24
Age (year)	63.05 ± 14.12	58.00 ± 16.26	66.42 ± 11.67
Sex (%)			
Male	13 (32.5)	2 (12.5)	11 (45.8)
Female	27 (67.5)	14 (87.5)	13 (54.2)
BMI (kg/m^2^)	26.91 ± 4.60	25.47 ± 3.50	27.86 ± 5.05
VAS	6.8 ± 0.91	6.88 ± 1.02	6.75 ± 0.85
ODI	36.03 ± 4.45	36.63 ± 4.59	35.63 ± 4.40

BMI: body mass index. VAS: visual analog scale. ODI: Oswestry disability index. FJ: facet joint. SIJ: sacroiliac joint.

**Table 2:** Improvements in VAS scores from baseline to follow-up visits estimated by GEE after adjusting for age, gender, and BMI.

Parameter	β	SE	95% Wald CI	*p*-Value
All patients (ref: baseline)				
1-week	−4.700	0.138	−4.970~−4.430	**<0.001**
1-month	−4.600	0.145	−4.884~−4.316	**<0.001**
3-month	−3.925	0.134	−4.188~−3.662	**<0.001**
6-month	−3.525	0.146	−3.811~−3.239	**<0.001**
Patients with FJ pain (ref: baseline)				
1-week	−4.500	0.234	−4.958~−4.042	**<0.001**
1-month	−4.625	0.263	−5.141~−4.109	**<0.001**
3-month	−3.813	0.268	−4.338~−3.287	**<0.001**
6-month	−3.500	0.265	−4.020~−2.980	**<0.001**
Patients with FJ and SIJ pain (ref: baseline)				
1-week	−4.833	0.163	−5.153~−4.514	**<0.001**
1-month	−4.583	0.166	−4.908~−4.258	**<0.001**
3-month	−4.000	0.132	−4.258~−3.742	**<0.001**
6-month	−3.542	0.166	−3.868~−3.215	**<0.001**

ref: reference. β: beta value. SE: standard error. CI: confidence interval. BMI: body mass index. FJ: facet joint. SIJ: sacroiliac joint. Bold fonts indicate statistical significance.

**Table 3:** Improvements in ODI score from baseline to follow-up visits estimated by GEE after adjusting for age, gender, and BMI.

Parameter	β	SE	95% Wald CI	*p*-Value
All patients (ref: baseline)				
1-week	−24.075	0.796	−25.634~−22.516	**<0.001**
1-month	−23.350	0.874	−25.064~−21.636	**<0.001**
3-month	−21.900	0.752	−23.375~−20.425	**<0.001**
6-month	−19.575	0.920	−21.378~−17.772	**<0.001**
Patients with FJ pain (ref: baseline)				
1-week	−22.813	1.367	−25.492~−20.133	**<0.001**
1-month	−22.938	1.614	−26.101~−19.774	**<0.001**
3-month	−21.563	1.375	−24.257~−18.868	**<0.001**
6-month	−19.813	1.680	−23.105~−16.520	**<0.001**
Patients with FJ and SIJ pain (ref: baseline)				
1-week	−24.917	0.924	−26.728~−23.106	**<0.001**
1-month	−23.625	0.979	−25.543~−21.707	**<0.001**
3-month	−22.125	0.853	−23.796~−20.454	**<0.001**
6-month	−19.417	1.046	−21.466~−17.376	**<0.001**

ref: reference. β: beta value. SE: standard error. CI: confidence interval. BMI: body mass index. FJ: facet joint. SIJ: sacroiliac joint. Bold fonts indicate statistical significance.

## Data Availability

Data supporting reported results can be requested from the first author.

## Supplementary Materials

The following are available online at https://www.mdpi.com/article/10.3390/diagnostics11101822/s1, Figure S1: The radiographs demonstrated the new strategy of diagnosis of low back pain following treatment with cooled radiofrequency ablation. The roentgenogram showed a modified Fortin finger test was adopted in a 67 years-old patient with persisted low back pain after fusion surgery (A). Clips were used to mark the pain source around right L34 and L45 facet joints. The ^99m^Tc-MDP SPECT/CT showed uptake activity around left sacroiliac joint (B and C). The following radiographs illustrated the procedure of cooled radiofrequency ablation in a C-arm fluoroscope suite (D). The intraoperative C-arm photos showed target positions of right L34 facet joint, medial branch (E) and left sacroiliac joint, S1 lateral branch (F); Figure S2 Box plots of VAS change scores from baseline to 1-week, 1-month, 3-month, and 6-month follow-up visits for all patients (a), patients with FJ pain (b), and patients with FJ and SIJ pain (c). *** indicates *p*-value < 0.001; Figure S3: Box plots of ODI change scores from baseline to 1-week, 1-month, 3-month, and 6-month follow-up visits for all patients (a), patients with FJ pain (b), and patients with FJ and SIJ pain (c). *** indicates *p*-value < 0.001.
