# Supplementary material for: A New Strategy for Rapid Diagnosis of the Source of Low Back Pain in Patients Scheduled to Undergo Treatment with Cooled Radiofrequency Ablation
Source: Diagnostics (Basel). 2021 Oct 1;11(10):1822. doi: 10.3390/diagnostics11101822 (PMC8534588; doi:10.3390/diagnostics11101822)
Supplement: Supplementary file 1 [file diagnostics-11-01822-s001.zip › diagnostics-1334314-supplementary.pdf]

Supplementary material

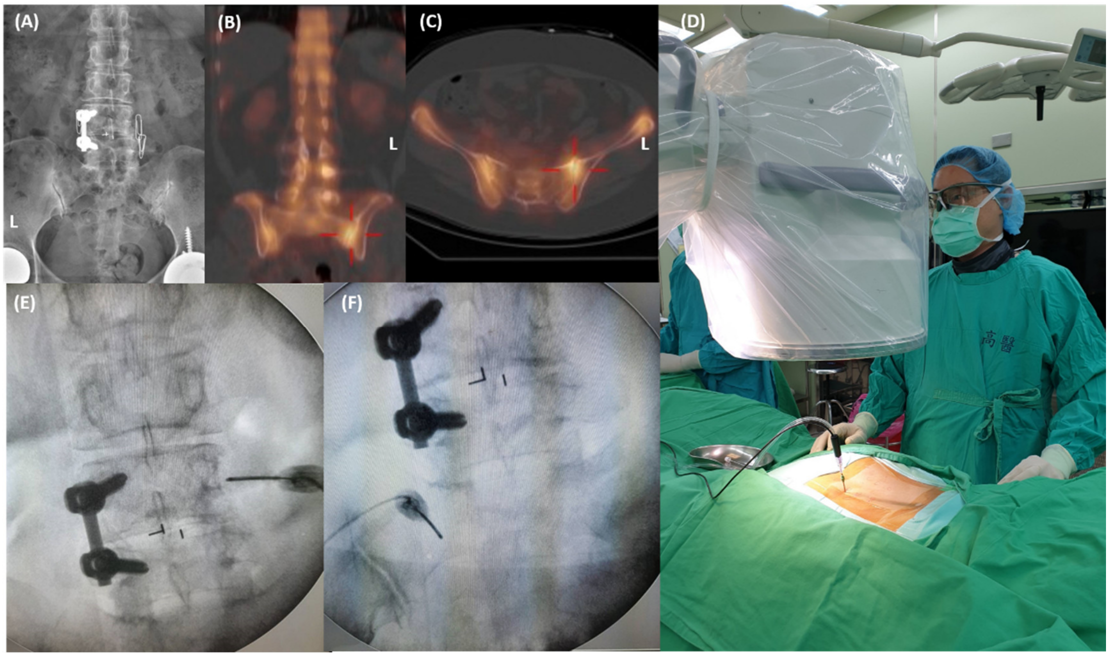

Figure S1. The radiographs demonstrated the new strategy of diagnosis of low back pain following treatment with cooled radiofrequency ablation. The roentgenogram showed a modified Fortin finger test was adopted in a 67 years-old patient with persisted low back pain after fusion surgery (A). Clips were used to mark the pain source around right L34 and L45 facet joints. The 99. mTc-MDP SPECT/CT showed uptake activity around left sacroiliac joint (B and C). The following radiographs illustrated the procedure of cooled radiofrequency ablation in a C-arm fluoroscope suite (D). The intraoperative C-arm photos showed target positions of right L34 facet joint, medial branch (E) and left sacroiliac joint, S1 lateral branch (F).

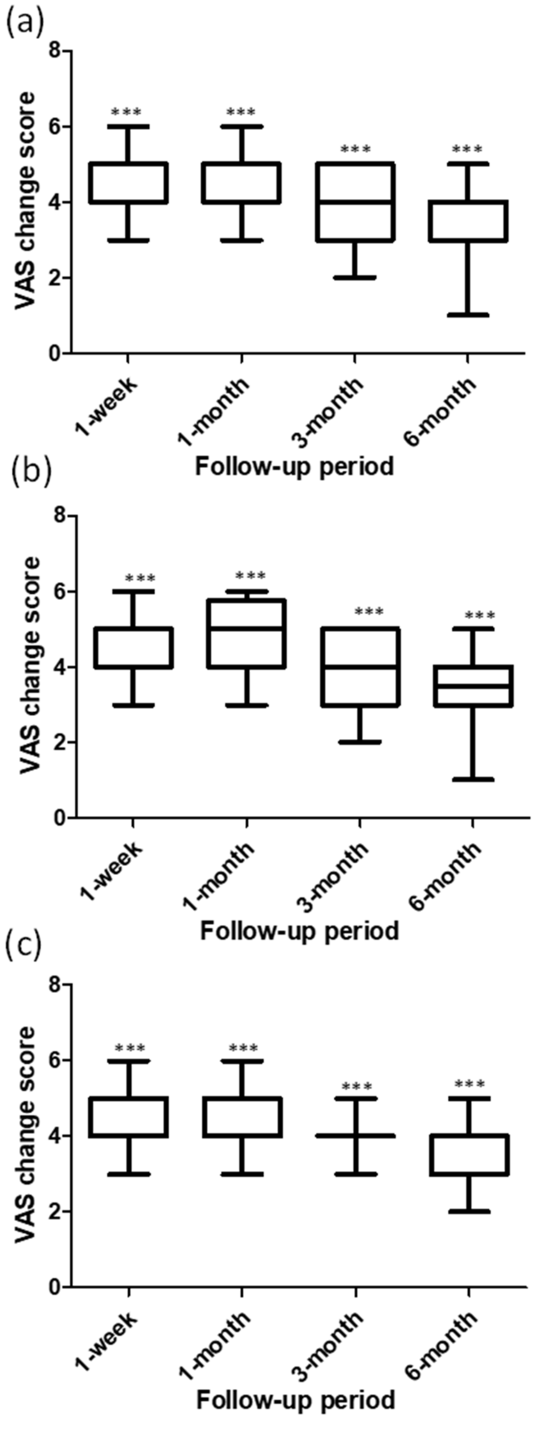

Figure S2. Box plots of VAS change scores from baseline to 1-week, 1-month, 3-month, and 6-month follow-up visits for all patients (a), patients with FJ pain (b), and patients with FJ and SIJ pain (c). *** indicates p-value < 0.001.

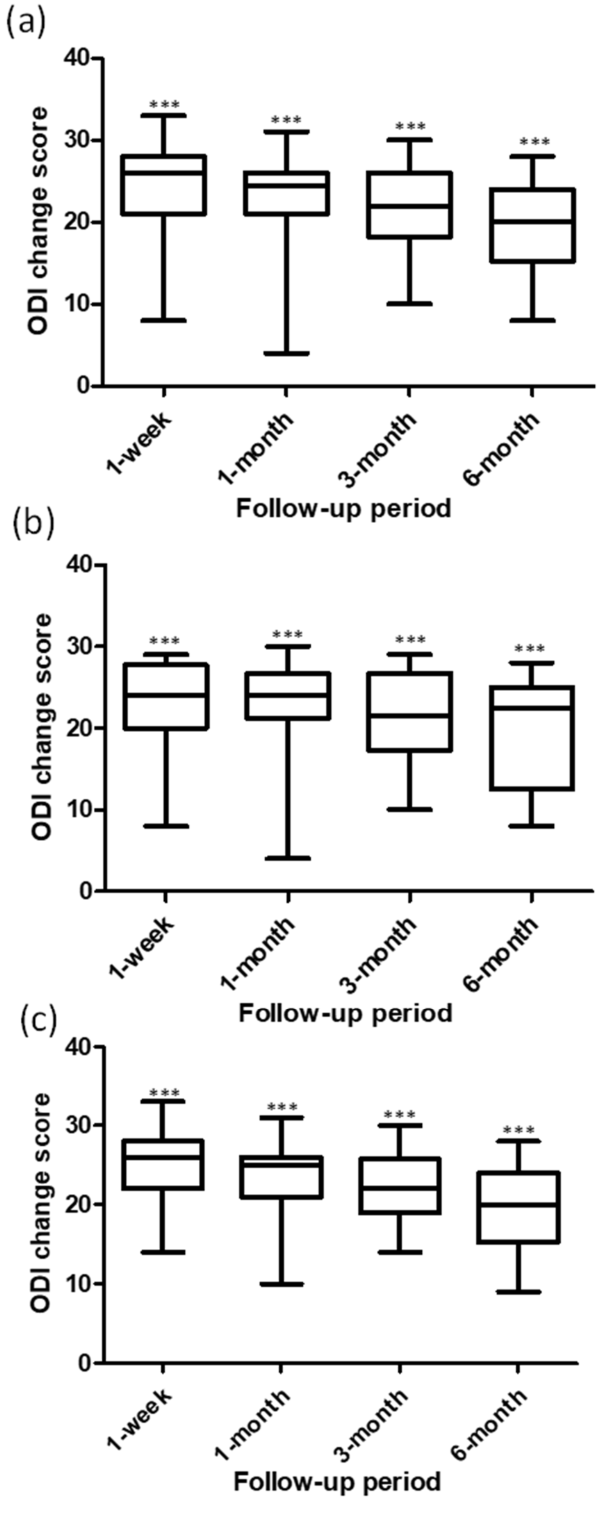

Figure S3. Box plots of ODI change scores from baseline to 1-week, 1-month, 3-month, and 6-month follow-up visits for all patients (a), patients with FJ pain (b), and patients with FJ and SIJ pain (c). *** indicates p-value < 0.001.
